# Prediction of immediate bleeding after cold snare polypectomy: A prospective observational study

**DOI:** 10.1097/MD.0000000000039597

**Published:** 2024-09-06

**Authors:** Shin Ju Oh, Yunho Jung, Young Hwangbo, Young Sin Cho, Il Kwun Chung, Chang Kyun Lee

**Affiliations:** a Department of Gastroenterology, Center for Crohn’s and Colitis, Kyung Hee University College of Medicine, Seoul, Korea; b Department of Internal Medicine, Soonchunhyang University College of Medicine, Cheonan, Korea; c Department of Preventive Medicine, Soonchunhyang University College of Medicine, Cheonan, Republic of Korea.

**Keywords:** cold snare polypectomy, colorectal polyp, postpolypectomy bleeding, small polyp

## Abstract

The risk factors for immediate post-polypectomy bleeding (IPPB) after cold snare polypectomy (CSP) are not well-known. We sought to define such risk factors and develop a predictive risk-scoring model. This prospective observational study included 161 polyps (4–9 mm in diameter) that were removed via CSP from 118 patients during the period from June to September 2019 in 2 tertiary hospitals. IPPB was defined as post-polypectomy bleeding within 24 hours or grade 3 or 4 intraprocedural bleeding requiring endoscopic hemostasis. IPPB incidences according to grade were 13.0% (21/161) (grade 3) and 0% (grade 4). Univariate analysis showed that the polyp size and morphology, as well as iatrogenic ulcer size and shape, were significantly associated with IPPB. Multivariate analysis showed that polyp size [6–9 mm vs 4–5 mm, odds ratio (OR) 3.72, 95% confidence interval (CI) 1.28–10.79], polyp morphology (polypoid vs non-polypoid, OR: 3.93, 95% CI: 1.22–12.64), and iatrogenic ulcer size (≥10 vs ≤ 9 mm, OR: 3.12, 95% CI: 1.04–9.38) were significantly associated with IPPB. We created a four-marker risk-scoring model to predict IPPB after CSP; we summed the points assigned for the 4 factors. At a cutoff of 2, the sensitivity was 85.7% and the specificity was 65.0%; at a cutoff of 3, the sensitivity was 65% and the specificity was 90.0%. Polyp size and morphology, as well as iatrogenic ulcer size and shape, were associated with IPPB after CSP. The four-marker risk-scoring model appears to effectively predict IPPB after CSP (Clinical Research Information Service: KCT0004375).

## 1. Introduction

Several endoscopic resection techniques are used to remove colorectal polyps. The paradigm is changing over time, reflecting the results of studies concerning the effectiveness of various techniques and improved instrumentation. The most important consideration is polyp size; diminutive/small polyps < 1 cm in diameter are commonly detected during colonoscopy. Currently, cold forceps polypectomy (CFP), cold snare polypectomy (CSP), and hot snare polypectomy (HSP) (with or without submucosal injection) are used to treat polyps < 1 cm in diameter; the chosen technique reflects the preference of the endoscopist who examines the polyps. CFP is commonly used to resect polyps ≤ 3 mm in diameter.^[1,2]^ The use of a jumbo biopsy forceps (compared to standard forceps) significantly increased the success rate of one-bite polypectomy and the complete resection rate of polyps 4 to 5 mm in diameter.^[3]^ However, CFP is generally not recommended for the removal of polyps ≥ 4 mm in size, regardless of whether jumbo biopsy forceps are employed, considering the limitation imposed by forceps cup size.^[4]^ HSP has been widely used to remove small polyps. However, the risks include delayed bleeding, post-polypectomy syndrome, and perforation caused by electrocautery-associated thermal injury.^[5]^

CSP is a simple and safe technique; a snare is used to mechanically transect polyps without the need for electrocautery. Several studies have reported more reliable results in terms of the removal of diminutive polyps than the results afforded by CFP, particularly when polyps are larger than 4 mm.^[1,6]^ Several meta-analyses found that the complete resection rate of CSP for small polyp was not inferior to that of HSP;^[7–9]^ and the delayed bleeding rates of CSP and HSP did not differ.^[7,8,10]^ CSP requires significantly less total colonoscopy and polypectomy times than does HSP because CSP does not use the submucosal injection and electrosurgical unit, thus saving time to inquiring about body attachments and applying a grounding pad.^[7]^ Thus, the European Society of Gastrointestinal Endoscopy^[11]^ and US Multi-Society Taskforce^[12]^ currently recommend CSP as the standard treatment for diminutive and small polyps. However, a recent survey of the colonic polypectomy preferences of Asian endoscopists revealed a greater preference for CFP than for CSP when encountering diminutive polyps, as well as a stronger preference for HSP than for CSP when treating small polyps.^[13]^ CSP is less favored than might be expected because of the potential for post-CSP immediate bleeding; a recent meta-analysis reported that the incidence of immediate post-polypectomy bleeding (IPPB) was higher after CSP than after HSP.^[10]^ However, it is not well known about the risk factors of IPPB after CSP. Thus, this study aimed to evaluate the risk factors for IPPB after CSP and to develop a predictive risk-scoring model.

## 2. Methods

### 2.1. Study population and design

**Figure 1.**
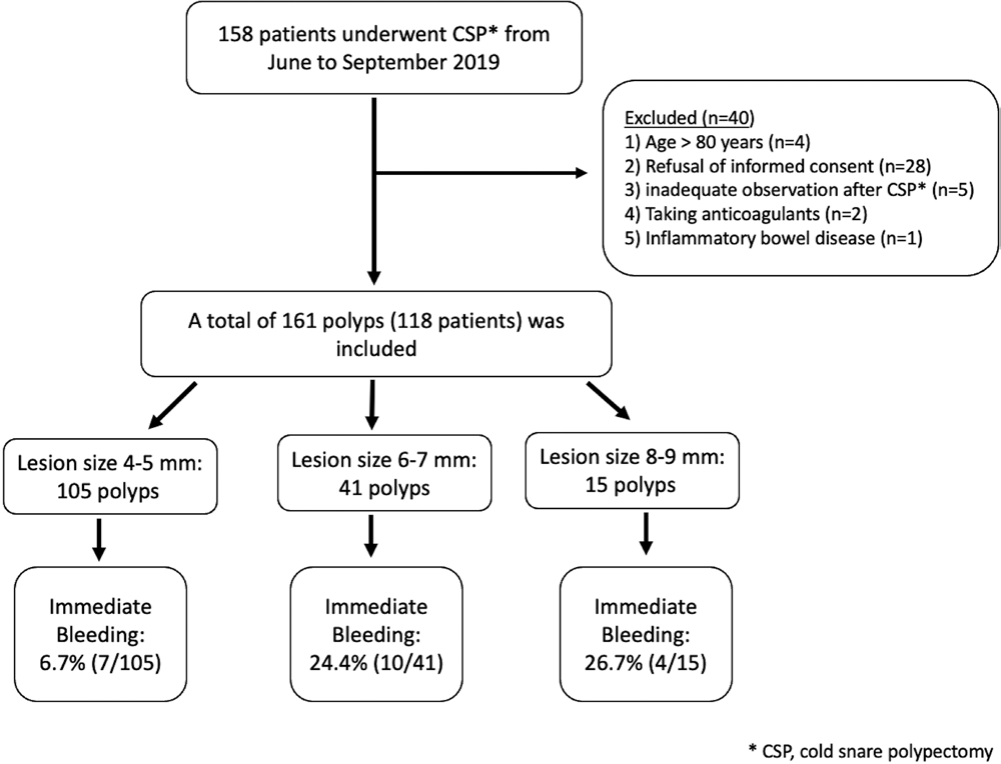
Flowchart of the study population.

The prospective observation study was conducted at 2 tertiary hospitals from June to September 2019, involving patients aged 20 to 80 who underwent a colonoscopy. Participants were eligible if they had 4 to 9 mm polyps detected and removed by cold snare polypectomy during the procedure. The exclusion criteria were: age <20 or >80 years; polyps suspected to be superficial or submucosal cancers based on the endoscopic features, chromoscopy, and narrow-band imaging; use of polypectomy techniques other than CSP such as HSP, EMR and modified EMR; performing submucosal injection during CSP; inadequate observation of iatrogenic ulcer bleeding status for 1 minute after polyp removal; use of antithrombotics or anticoagulants within the prior 3 months or a history of inflammatory bowel disease; and refusal to provide informed consent for participation in the study (Fig. 1).

### 2.2. CSP equipment and procedure

All procedures were performed by experienced endoscopists who were affiliated with the hospital gastroenterology departments. High-definition colonoscopes (series 260 and 290; Olympus America, Center Valley, PA) were used in all procedures; all polyps were removed by employing Exacto cold snares (US Endoscopy, Mentor, OH). Polyp size was estimated by reference to the size of the open snare loop or the snare catheter. CSP was performed as previously described.^[14]^ Each polyp was positioned in the 5 to 6 o’clock direction of the endoscopic channel; ≥1 to 2 mm of normal tissue around the polyp was grasped when fully opening the snare. The snare was closed via gentle forward pressure on the snare catheter, and the polyps were transected without tenting. After polyp retrieval via the suction channel into a trap, all polypectomy sites were carefully screened in terms of residual polyp status.

### 2.3. Definitions/classifications of various factors

IPPB was divided into intra- and post-procedural immediate bleeding (depending on colonoscope withdrawal status). The degree of intraprocedural bleeding was classified into 4 categories based on previous literature^[15]^: grade 1, spontaneous hemostasis within 1 minute; grade 2, continuous but decreased oozing over 1 minute; grade 3, continuous oozing over 1 minute; and grade 4, active spurting. According to the need for endoscopic hemostasis, grade 1 and 2 cases formed the non-bleeding group, while grade 3 and 4 cases the bleeding group. IPPB was defined as the occurrence of post-polypectomy bleeding within 24 hours or grade 3 or 4 of intraprocedural bleeding requiring endoscopic hemostasis. Delayed post-polypectomy bleeding (DPPB) was defined as bleeding that occurred ≥24 hours after the procedure.

**Figure 2.**
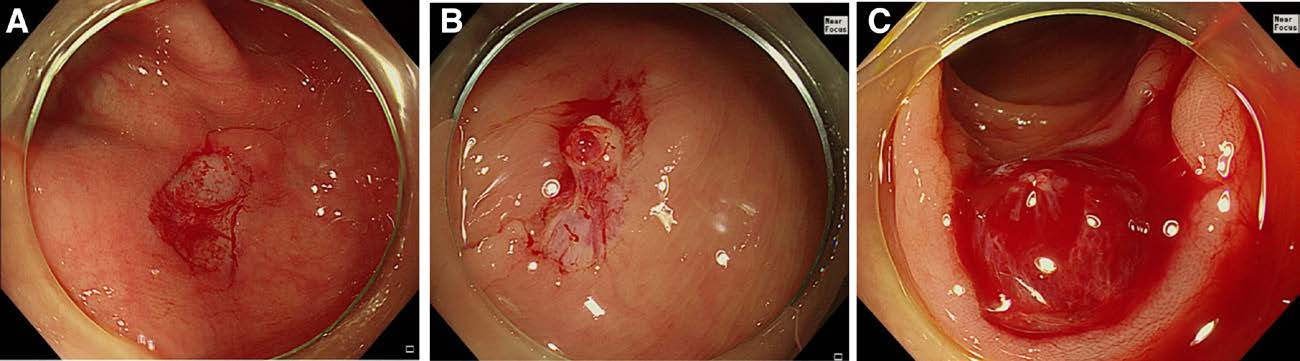
Endoscopic classification of iatrogenic ulcers according to shape. (A) Type I (flat); (B) type II (focal defect protrusion); (C) type III (diffuse defect protrusion).

Cold snare defect protrusions include muscularis mucosa and submucosa tissues.^[16]^ We classified the iatrogenic ulcer shape into 3 categories according to the extent of defect protrusion: type I (flat; no protrusion); type II (focal defect protrusion within 1/3 of the defect); and type III (diffuse defect protrusion over >1/3 of the defect) (Fig. 2).

### 2.4. Outcome parameters and subgroup analysis

We carefully evaluated patient age, sex, and comorbidities; polyp location, size, morphology, and pathology; and iatrogenic ulcer size, shape, and hematoma status. We sought associations between these factors and IPPB.

### 2.5. Ethical approval and statistical analysis

This study protocol was approved by the Institutional Ethics Committees of both participating hospitals (IRB number SCHCA 2018-12-017, KHUH 2018-11-066) and is registered in the Clinical Research Information Service (https://cris.nih.go.kr/cris/en/index.jsp) (KCT0004375). Informed consent was obtained from all patients before enrollment. The incidence of IPPB after CSP has been reported as 3.3% to 19.7%,^[9,10,17]^ but the risk factors for IPPB after CSP are not well-known. Therefore, we estimated a sample size ≥ 146 was required to achieve a statistical power of 80% at 5% significance, assuming that the IPPB incidence after CSP differed by 15% between patients with small (6–9 mm) and diminutive (4–5 mm) polyps. Anticipating a 10% dropout rate, we sought to enroll 162 cases. Differences in categorical variables between the bleeding and non-bleeding groups were assessed using the chi-squared test. The development of predictive model for IPPB was performed in multiple steps. First, univariate logistic regression was conducted for each variable. And variables with *P* < .05 were selected for the final predictive model for IPPB. Second, we developed 2 predictive models. The first one is logistic regression predictive model which was established using the β-coefficients of the final multivariate logistic regression.^[18]^ The other is risk score model based on risk score for IPPB which were points assigned using the final logistic regression model as previously described.^[19]^ The purpose of risk score model is to make complex statistical model (logistic regression predictive model) simple and useful to clinical practitioners. Model discrimination was assessed by evaluating the area under the curve (AUC) of the receiver operator characteristic (AUC) curve and model calibration was evaluated with calibration plots. The risk scores were subjected to receiver operating characteristic analysis to assess their utilities in terms of IPPB prediction, then to determine optimal cutoffs. Log-binomial regression analysis was used to estimate IPPB relative risks with 95% confidence intervals (CIs); we compared risk scores to the reference values (≤1). A *P*-value <.05 was considered statistically significant. All analyses were performed using STATA software (ver. 14.0; StataCorp, College Station, TX).

## 3. Results

### 3.1. Clinical characteristics and outcomes

**Figure 3.**
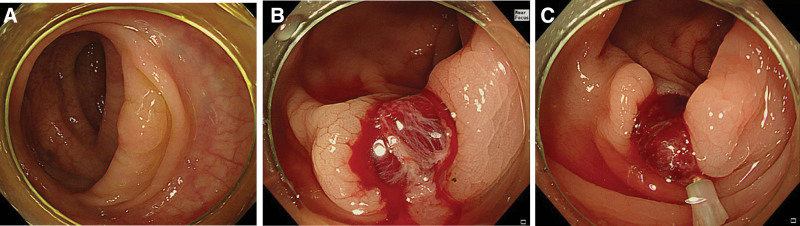
(A) A 5-mm-diameter polyp located in the sigmoid colon. (B) Continuous oozing over 1 minute after polypectomy. (C) Performance of endoscopic hemostasis.

In total, 161 polyps 4 to 9 mm in diameter were successfully removed from 118 patients. The mean patient age was 61.2 ± 11.8 years; 63.6% were men and 36.4% were women (Table 1). The *en bloc* resection rate was 100% (161/161); the fly-away and specimen losses were both 1.2% (2/161). The mean polyp and iatrogenic ulcer sizes were 5.3 ± 1.4 and 6.4 ± 2.6 mm, respectively; iatrogenic ulcers 5 to 9 mm in size were most common. In terms of iatrogenic ulcer shapes, 69% (111/161) were type I, 21.1% (34/161) were type II, and 9.9% (16/161) were type III. The intraprocedural bleeding incidences were 32.3% (52/161) for grade 1, 28.6% (46/161) for grade 2, 13.0% (21/161) for grade 3, and 0% for grade 4. Endoscopic hemostasis was required by 12.4% (20/161) of all cases and 16.8% (20/119) of cases with intraprocedural bleeding (Fig. 3). Hemoclips were used to endoscopic hemostasis method in all cases and; the mean number of hemoclips used per case was 1.5 ± 0.6. We encountered no case of post-procedural immediate bleeding, DPPB, or perforation (Table 2).

**Table 1:** Baseline characteristics of patients and polyps.

Patients (n = 118)	N (%)
Age, mean ± SD	61.2 ± 11.8
Sex	
Male	75 (63.6)
Female	43 (36.4)
Indication	
Screening	57 (48.3)
Surveillance	61 (51.7)
Hypertension	44 (37.3)
Diabetes	20 (17.0)
**Polyps (n = 161**)	**N (%**)
Location	
Right colon	88 (54.6)
Left colon	65 (40.4)
Rectum	8 (5.0)
Size (mm)	
4–5	105 (65.2)
6–7	41 (25.5)
8–9	15 (9.3)
Morphology	
Flat	77 (47.8)
Sessile	82 (50.9)
Pedunculate	2 (1.3)
Pathology	
Serrated polyp	36 (22.8)
Adenoma	122 (77.2)
Cancer	0

**Table 2:** Overall results of cold snare polypectomy.

Results of cold snare polypectomy (n = 161)	N (%)
En bloc resection	161 (100)
Flay away of specimen	2 (1.2)
Loss of specimen	2 (1.2)
Difficult to cut	8 (5.0)
Iatrogenic ulcer size (mm)	
≤5	41 (25.5)
6–9	94 (58.4)
≥10	26 (16.1)
Iatrogenic ulcer shapes	
Type I (flat)	111 (69.0)
Type II (focal defect protrusion)	34 (21.1)
Type III (diffuse defect protrusion)	16 (9.9)
Hematoma	
Yes	13 (8.1)
No	148 (91.9)
Grade of Intraprocedural bleeding*	
Grade 1	52 (32.3)
Grade 2	46 (28.6)
Grade 3	21 (13.0)
Grade 4	0
Endoscopic hemostasis	
Yes	20 (12.4)
No	141 (87.6)
Endoscopic hemostasis methods	
Hemoclips	20 (100%)
Forceps coagulation	0
Argon plasma coagulation	0
Number hemoclips used per case (± standard deviation)	1.5 ± 0.6
Post-procedural immediate bleeding	0
Delayed post polypectomy bleeding	0
Perforation	0

* Grade 1, spontaneous hemostasis within 1 minute; grade 2, continuous but decreased oozing over 1 minute; grade 3, continuous oozing over 1min; and grade 4, active spurting.

### 3.2. Factors associated with IPPB

Univariate analysis showed that polyp size (4–5 mm: 6.7%, 6–7 mm: 24.4%, and 8–9 mm: 26.7%; *P* = .004) and iatrogenic ulcer size (≤ 5 mm: 12.2%, 6–9 mm: 7.5%, and ≥10 mm: 34.6%; *P* = .001) were significantly associated with IPPB. Iatrogenic ulcer shape (type I: 8.1%, type II: 23.5%, and type III: 25.0%; *P* = .021) and polyp morphology (non-polypoid 6.5% vs polypoid 19.1%; *P* = .018) were also significantly associated with IPPB. However, polyp location, pathological findings, and hematoma status were not significantly associated with IPPB (Table 3). Multivariate analysis showed that polyp size (6–9 mm vs 4–5 mm, odds ratio [OR]: 3.72, 95% CI: 1.28–10.79; *P* = .016); polyp morphology (polypoid vs non-polypoid, OR: 3.93, 95% CI: 1.22–12.64; *P* = .022); and iatrogenic ulcer size (≥10 mm vs ≤9 mm, OR: 3.12, 95% CI: 1.04–9.38; *P* = .042) were significantly associated with IPPB (Table 4).

**Table 3:** Univariate analysis of factors associated with immediate bleeding after cold snare polypectomy.

	Total (n, %)	Non-bleeding group* (N = 140)	Bleeding group† (N = 21)	*P*-value
*Polyp location* (*n*, %)				.475
Right colon	88 (54.6)	74 (84.1)	14 (15.9)	
Left colon	65 (40.4)	59 (90.8)	6 (9.2)	
Rectum	8 (5.0)	7 (87.5)	1 (12.5)	
*Polyp size* (*mm*)				.004
4–5	105 (65.2)	98 (93.3)	7 (6.7)	
6–7	41 (25.5)	31 (75.6)	10 (24.4)	
8–9	15 (9.3)	11 (73.3)	4 (26.7)	
*Polyp morphology*				.018
Non polypoid (flat)	77 (47.8)	72 (93.5)	5 (6.5)	
Polypoid (Sessile, pedunculate)	84 (52.2)	68 (80.9)	16 (19.1)	
*Polyp pathology*				.904
Serrated polyp	36 (22.8)	31 (86.1)	5 (13.9)	
Adenoma	122 (77.2)	106 (86.9)	16 (13.1)	
*Iatrogenic ulcer size* (*mm*)				.001
≤ 5	41 (25.5)	36 (87.8)	5 (12.2)	
6–9	94 (58.4)	87 (92.6)	7 (7.5)	
≥ 10	26 (16.1)	17 (65.4)	9 (34.6)	
*Iatrogenic ulcer shapes*				.021
Flat	111 (68.9)	102 (91.9)	9 (8.1)	
Focal submucosal protrusion	34 (21.1)	26 (76.5)	8 (23.5)	
Diffuse submucosal protrusion	16 (10.0)	12 (75.0)	4 (25.0)	
*Hematoma*				.794
Yes	13 (8.1)	11 (84.6)	2 (15.4)	
No	148 (91.9)	129 (87.2)	19 (12.8)	
*Endoscopic hemostasis*				<.001
Yes	20 (12.4)	4 (20.0)	16 (80.0)	
No	141 (87.6)	136 (96.5)	5 (3.5)	

* Non-bleeding group included non-bleeding after polypectomy, grade 1 and grade 2 of intraprocedural bleeding.

† Bleeding group (Grade 3) included grade 3 and grade 4 of intraprocedural bleeding.

**Table 4:** Multivariate logistic regression analysis and points assigned for immediate bleeding after cold snare polypectomy.

Factors		Odds ratio	95% confidential interval	*P*-value	Points assigned
Polyp size	4–5 mm		Reference		
	6–9 mm	3.72	1.28–10.79	.016	1
Polyp morphology	Non-polypoid		Reference		
	Polypoid	3.93	1.22–12.64	.022	1
Iatrogenic ulcer size	≤9 mm		Reference		
	≥10 mm	3.12	1.04–9.38	.042	1
Iatrogenic ulcer shapes	flat		Reference		
	Focal or diffuse defect protrusion	2.41	0.84–6.90	.100	1

### 3.3. Development of predictive model and calculation of risk scores of IPPB

**Figure 4.**
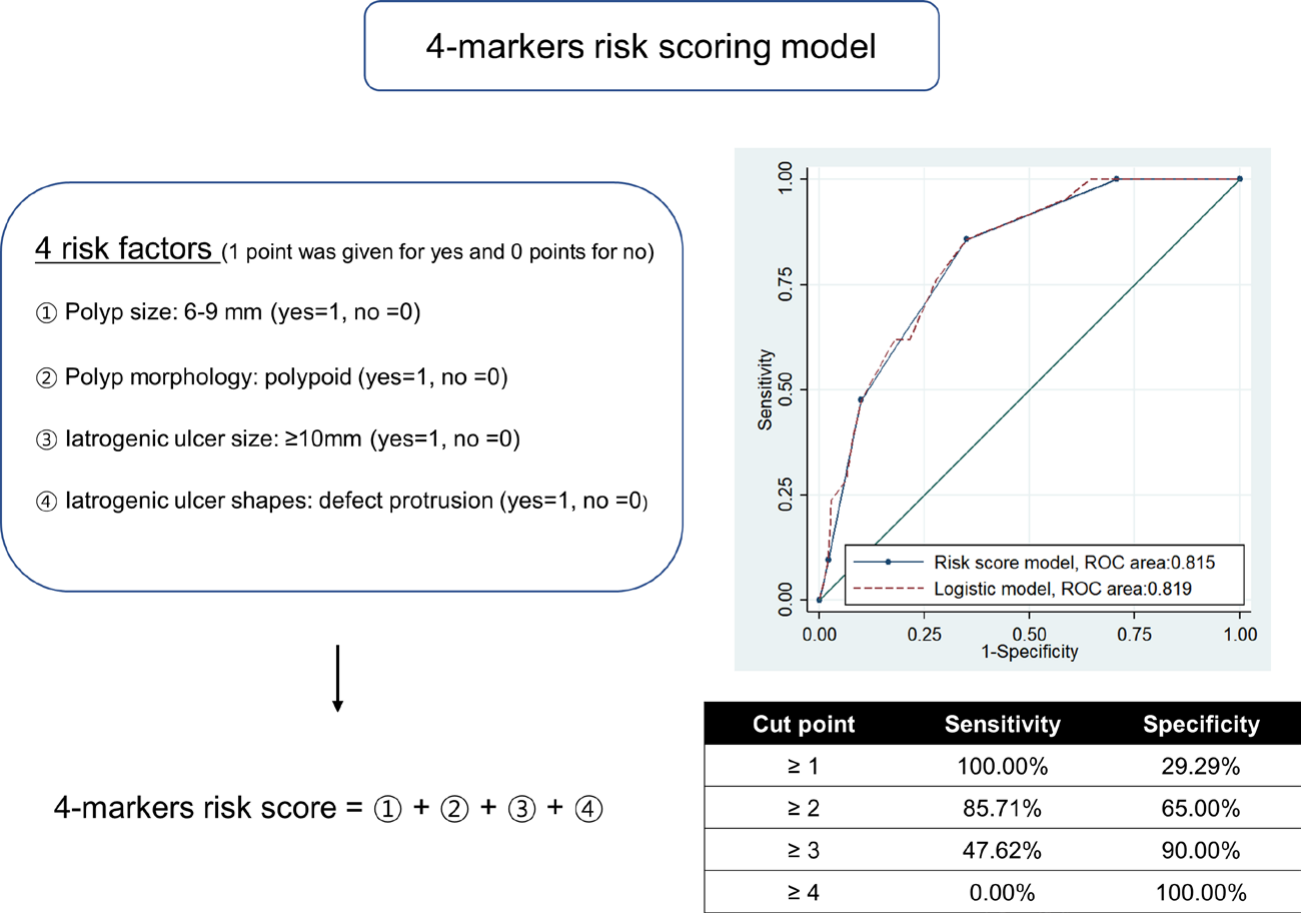
The four-marker risk scoring model with corresponding receiver operating characteristic curves (ROC) curves of logistic regression model and risk score model.

**Figure 5.**
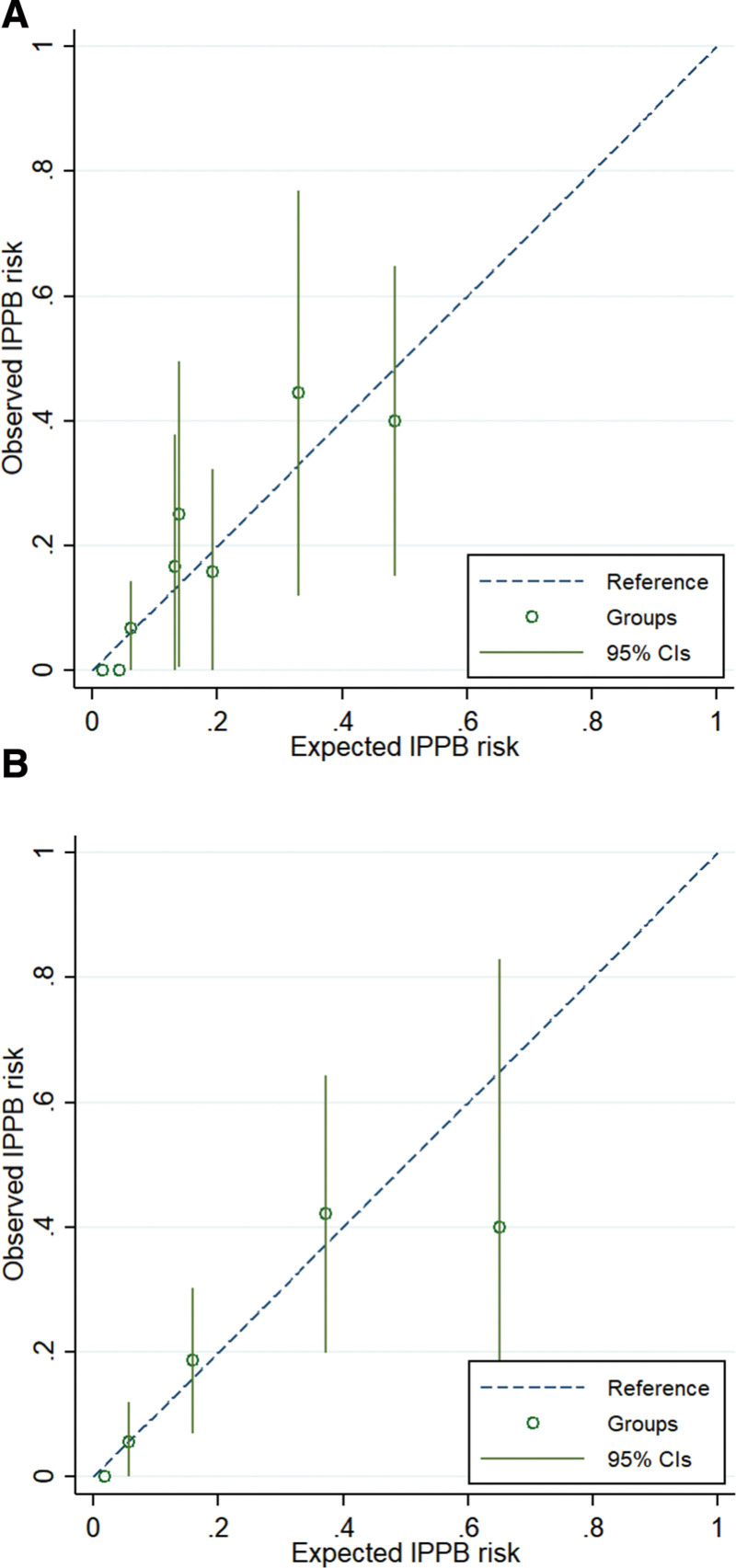
Calibration plots showing observed and predicted risks across deciles using (A) logistic regression model, and (B) risk score model.

Univariate logistic regression was performed, and 4 variables with *P* < .05 entered final multivariate logistic regression (Table 3): polyp size, polyp morphology, iatrogenic ulcer size, and iatrogenic ulcer shape. Based on the β coefficients of the final logistic regression, points were assigned to each of the 4 predictors, so that ranges of total risk score was from 0 to 4 (Table 4). The AUC of the risk score model for IPPB (0.815, 95% CI 0.745–0.871) was very similar with the one using logistic regression predictive model (0.819, 95% CI 0.752–0.876) (Fig. 4), and both models were well-calibrated (Fig. 5). To simplify the assessment of IPPB risk and assist clinicians in their decision-making process regarding treatment, we created a four-marker risk scoring system to predict IPPB after CSP, as follows: (1) polyp size 6–9 mm (yes = 1, no = 0); (2) polyp morphology: polypoid (yes = 1, no = 0); (3) iatrogenic ulcer size ≥ 10 mm (yes = 1, no = 0); and (4) iatrogenic ulcer shape: defect protrusion (yes = 1, no = 0) and we summed the assigned points. A cutoff of 2 afforded a sensitivity of 85.7% and a specificity of 65.0%; a cutoff of 3 afforded a sensitivity of 65% and a specificity of 90.0%. (Fig. 4). The directly estimated relative risk of IPPB when comparing patients with risk score of 2 versus those who have risk scores ≤1 was 5.8 (95% CI: 1.6–20.9). For the groups with risk scores of 3 and 4, the relative risks were 13.2 (95% CI: 3.8–45.2) and 12.5 (95% CI: 2.7–58.9), respectively (Table 5).

**Table 5:** Estimated relative risks of IPPB according to risk scores.

Risk score*	Total (n)	Non-bleeding group† (n, %)	Bleeding group‡ (n, %)	Relative risk (95% CI)
0–1	94	91 (96.8)	3 (3.2)	Reference
2	43	35 (81.4)	8 (18.6)	5.8 (1.6–20.9)
3	19	11 (57.9)	8 (42.1)	13.2 (3.8–45.2)
4	5	3 (60.0)	2 (40.0)	12.5 (2.7–58.9)

* The risk score was calculated as the sum of the values of 4 factors such as polyp size (6–9 mm; yes = 1, no = 0) and morphology (polypoid; yes = 1, no = 0), iatrogenic ulcer size (≥10mm; yes = 1, no = 0), and shapes (defect protrusion; yes = 1, no = 0).

† Non-bleeding group included non-bleeding after polypectomy, grade 1 and grade 2 of intraprocedural bleeding.

‡ Bleeding group (Grade 3) included grade 3 and grade 4 of intraprocedural bleeding.

## 4. Discussion

To our knowledge, this is the first study to evaluate a scoring system for predicting IPPB following CSP, based on prospectively collected data on variables and outcomes in real clinical patients. The overall IPPB rate was 73.9% (119/161) and endoscopic hemostasis was required by 12.4% (20/161) of all cases. Factors such as polyp size, polyp morphology, and iatrogenic ulcer sizes were found to significantly influence the risk of IPPB. Specifically, larger polyps (6–9 mm) and larger iatrogenic ulcers (≥10 mm) increased the likelihood of IPPB, as did polypoid morphology. We developed a predictive model and created a risk scoring system based on these variables and found that a risk score of 2 or more was associated with an increased risk of IPPB. This scoring system provides a practical tool for clinicians to assess and manage the risk of IPPB in patients undergoing CSP.

In most cases, IPPB can generally be managed effectively with endoscopic techniques when recognized and addressed promptly. It often occurs within minutes following a polypectomy, and methods such as epinephrine injection or clipping during colonoscopy can control it effectively. Consequently, IPPB is deemed less clinically significant than DPPB, mainly because it can be quickly identified and managed. In contrast, DPPB is associated with relatively greater risks of hemodynamic instability, hospitalization, and a need for blood transfusion; DPPB is usually detected late and the time required to stop bleeding is longer than the time needed to control IPPB.^[20]^ CSP is associated with a low incidence of DPPB^[7]^ because CSP does not use electrocautery; contrary to HSP, there is no possibility that electrical damage will progress over time.^[21]^ Furthermore, perforation during CSP is almost negligible since transecting the muscularis propria is nearly impossible.^[22]^ Indeed, we countered no DPPB nor perforation.

However, the rapid recognition and management of IPPB remain crucial. Patients at high risk for IPPB, might require extended post-procedure monitoring, including checks for vital signs, signs of continued bleeding, and overall stability before discharge. Additionally, exploring risk factors for immediate bleeding and developing methods to mitigate these risks could change the clinical strategy of endoscopic polypectomy.^[23]^ Pre-procedural measures such as adjusting anticoagulant and antiplatelet therapy in light of the recently updated consensus and in consultation with prescribing physicians, can be crucial.^[24]^ Additionally, employing meticulous technique to ensure complete hemostasis, such as prophylactic clipping, applying hemostatic agents including hemostatic powder, or using thermal coagulation, especially in high-risk patients, can prevent IPPB.^[20,25]^ Studies focusing on the relationships between procedural variables, patient characteristics, and the incidence of IPPB are invaluable for advancing clinical practice.

CSP is simple, safe, and faster than other polypectomy techniques.^[7]^ CSP has recently been recommended by European Society of Gastrointestinal Endoscopy and US Multi-Society Taskforce to treat polyps <10 mm in diameter.^[11,12]^ However, in a recent survey of Asian endoscopists, only 16.9% (26/154) favored CSP to treat diminutive polyps and only 31.2% chose CSP (48/154) to treat small polyps, perhaps because the guidelines do not reflect the real-world experiences of practitioners.^[13]^ The reason for these results is presumed that IPPB is more common after CSP than after the application of other polypectomy techniques.

The incidence of IPPB after CSP is 3% to 20%.^[9,10,17]^ The variation is probably attributable to differences in the sizes and characteristics of polyps and iatrogenic ulcers, as well as the various definitions of IPPB used. Immediate bleeding after CSP is mostly capillary bleeding; thus, it generally ceases with time and does not require endoscopic hemostasis. However, it is difficult to make an intraprocedural determination of the need for endoscopic hemostasis; most endoscopists prefer to stop bleeding immediately. In the present study, the incidence of IPPB recognized by observation for 1 minute was 13% (21/161). This is the time allowed in real clinical practice. Accordingly, we analyzed factors that affected IPPB.

Polyp size (>1 cm), morphology (pedunculated type), and location (right colon) have been reported as risk factors for post-polypectomy bleeding.^[15,26,27]^ Although few studies have explored IPPB risk factors, one study reported that risk factors for IPPB after HSP included older age (≥65 years), comorbid cardiovascular or chronic renal disease, anticoagulant use, polyp size > 1 cm, a pedunculated polyp or a laterally spreading tumor, and poor bowel preparation.^[15]^

Although all polyps were <1 cm in size, polyp size [6–9 vs 4–5 mm, OR: 3.72, 95% CI: 1.28–10.79; *P* = .016] was a significant risk factor for IPPB after CSP. Polyp morphology (polypoid vs non-polypoid) was also significantly associated with IPPB (OR: 3.93, 95% CI: 1.22–12.64; *P* = .022). However, in contrast to the results of previous studies,^[26,28]^ we found no significant relationship between polyp location and the risk of IPPB. Iatrogenic ulcers were morphologically classified into 3 types according to the extent of cold snare defect protrusions. This classification was not performed in previous studies. We prospectively evaluated IPPB development after CSP. As the defect protrusion became more diffuse, the risk of IPPB increased (type I: 8.1%, type II: 23.5%, and type III: 25.0%; *P* = .021). Iatrogenic ulcer size was carefully measured because it was previously identified as a risk factor for post-polypectomy bleeding.^[29]^ We found that ulcer size (≥10 vs ≤9 mm, OR: 3.12, 95% CI: 1.04–9.38; *P* = .042) was significantly associated with IPPB, probably because a larger ulcer led to greater likelihood of damage to the capillaries and penetrating vessels, thereby increasing the bleeding risk.

We developed a four-marker risk scoring model for IPPB after CSP. One point each is assigned to polyp size and morphology, as well as iatrogenic ulcer size and shape. A cutoff of 2 afforded a high sensitivity of 85.7%, while a cutoff of 3 afforded a high specificity of 90.0%. Compared to patients with scores ≤ 1, patients with scores of 2 had a 5.8-fold greater risk of IPPB; patients with scores of 3 and 4 had 13.2- and 12.5-fold greater risks, respectively. We encountered no post-procedural IPPB or DPPB, probably because endoscopic hemostasis was performed on patients with grade 3 IPPB and CSP causes minimal damage to the submucosal layer.

Our work had some limitations. The sample size was relatively small and iatrogenic ulcer shape evaluation was subjective. However, the possible shapes differed markedly, and shape may thus be a valid criterion.

To our knowledge, this is the first prospective study to define risk factors for IPPB after CSP in real-world clinical practice. It is important to observe the size and shape of iatrogenic ulcers. The four-marker risk scoring model simply and effectively predicted IPPB after CSP in clinical practice. We hope this study could give clinicians some aids in their decision making process regarding treatment. However, this model could be further developed and externally validated using a large scale multicenter study.

In conclusion, polyp size and morphology, as well as iatrogenic ulcer size and shape, were associated with IPPB after CSP. The four-marker risk scoring model appears to effectively predict IPPB after CSP.

## Author contributions

**Conceptualization:** Shin Ju Oh, Yunho Jung, Young Hwangbo, Young Sin Cho, Il Kwun Chung, Chang Kyun Lee.

**Data curation:** Shin Ju Oh, Yunho Jung, Young Hwangbo, Young Sin Cho, Il Kwun Chung, Chang Kyun Lee.

**Formal analysis:** Yunho Jung, Young Hwangbo.

**Methodology:** Yunho Jung, Young Hwangbo.

**Writing – original draft:** Shin Ju Oh, Yunho Jung.

**Writing – review & editing:** Shin Ju Oh, Yunho Jung, Chang Kyun Lee.
